# A Combined Acceptor Photobleaching and Donor Fluorescence Lifetime Imaging Microscopy Approach to Analyze Multi-Protein Interactions in Living Cells

**DOI:** 10.3389/fmolb.2021.635548

**Published:** 2021-05-14

**Authors:** Robert Eckenstaler, Ralf A. Benndorf

**Affiliations:** Institute of Pharmacy, Martin-Luther-University Halle-Wittenberg, Halle, Germany

**Keywords:** multiple protein–protein interactions, three-fluorophore FRET, 3-way FRET, acceptor photobleaching, FLIM

## Abstract

Protein–protein interaction studies often provide new insights, i.e., into the formation of protein complexes relevant for structural oligomerization, regulation of enzymatic activity or information transfer within signal transduction pathways. Mostly, biochemical approaches have been used to study such interactions, but their results are limited to observations from lysed cells. A powerful tool for the non-invasive investigation of protein–protein interactions in the context of living cells is the microscopic analysis of Förster Resonance Energy Transfer (FRET) among fluorescent proteins. Normally, FRET is used to monitor the interaction state of two proteins, but in addition, FRET studies have been used to investigate three or more interacting proteins at the same time. Here we describe a fluorescence microscopy-based method which applies a novel 2-step acceptor photobleaching protocol to discriminate between non-interacting, dimeric interacting and trimeric interacting states within a three-fluorophore setup. For this purpose, intensity- and fluorescence lifetime-related FRET effects were analyzed on representative fluorescent dimeric and trimeric FRET-constructs expressed in the cytosol of HEK293 cells. In particular, by combining FLIM- and intensity-based FRET data acquisition and interpretation, our method allows to distinguish trimeric from different types of dimeric (single-, double- or triple-dimeric) protein–protein interactions of three potential interaction partners in the physiological setting of living cells.

## Introduction

Förster or fluorescence resonance energy transfer (FRET) is a physical effect between two interacting fluorophores called the FRET donor and the FRET acceptor (Clegg, 1995; Bajar et al., 2016). During this interaction, a fraction of the energy of the donor’s excited state is transferred to the acceptor in a radiation-free manner. This energy transfer requires, among others, the spectral overlap of the emission spectrum of the donor with the excitation spectrum of the acceptor and a very close proximity of the donor and the acceptor in the nanometer range. Distances above the Förster radius of a specified FRET pair drastically reduce the energy transfer, making FRET a powerful tool in fluorescence microscopy to study interactions and conformational changes of proteins that cannot actually be resolved with fluorescence microscopy itself (Holden et al., 2010). In this regard, the occurrence of FRET causes three major changes in the spectral properties of the donor and the acceptor that are utilized to monitor FRET by fluorescence microscopy (Day and Davidson, 2012). First, there is a reduction in donor fluorescence intensity and the reversal of this effect is used to detect FRET in acceptor photobleaching approaches. Second, there is a reduction of the donor fluorescence lifetime which can be monitored by fluorescence lifetime imaging (FLIM). And third, there is sensitized emission of the acceptor while exciting the donor, a phenomenon that is measured by corrected FRET or spectral imaging approaches. Traditionally, these approaches have been developed to study FRET between two fluorophores and therefore only provide insights into the interaction of two proteins. In recent years, however, FRET analysis techniques have been further developed to enable the simultaneous measurement of multiple protein interactions using detection methods with advanced FRET corrections for up to three fluorophores (Galperin et al., 2004; Pauker et al., 2012; Fried et al., 2014) or linear unmixing of spectral components of the multiple fluorophores involved (Sun et al., 2010; Wallrabe et al., 2012; Hoppe et al., 2013; Scott and Hoppe, 2016). In addition to fluorescence microscopy, fluorescence spectroscopy (Lee et al., 2007; Aneja et al., 2008; Chung et al., 2017; Yoo et al., 2020) and flow cytometry (Fábián et al., 2013) have been used to analyze multi-FRET scenarios. So far, most microscopy studies have focused on detecting sensitized emission of the acceptor to study FRET among multiple fluorophores. This approach seems to be advantageous for the imaging of living cells, since much faster acquisition rates can be achieved compared to fluorescence lifetime or acceptor photobleaching set-ups. In addition, the approach largely preserves the fluorescence of the donor and the acceptor allowing for repetitive FRET detection which is impossible, e.g., in acceptor photobleaching approaches. However, measurement of FRET-induced sensitized emission faces a common problem that already exists in a two-fluorophore configuration, namely bleed-through of the donor fluorescence in the spectral range of the acceptor and direct acceptor excitation during donor excitation (Kaminski et al., 2014). Therefore, to visualize the sensitized emission of the acceptor, either image corrections or spectral separations are needed to obtain the pure sensitized emission component within the image. In a three-fluorophore configuration, the presence of a third fluorophore enhances the complexity even further. Consequently, cost-intensive microscopic setups for spectral imaging and complex linear unmixing algorithms or the acquisition of perfectly aligned multi-color images and rather complex image corrections between them are necessary to ensure valid results. Moreover, the apparent FRET efficiencies/FRET indices obtained by these methods are mostly not comparable to those from other studies due to the considerable variety of normalization methods available for corrected FRET data. Here we report a method to distinguish non-interacting, dimeric and trimeric interacting proteins in living cells using a two-step acceptor photobleaching protocol for a three-fluorophore setup. This method provides a snapshot of the state of protein interactions and cannot be used for repeated measurements on the same cell. However, it can be easily used with common setups for confocal laser-scanning microscopy and does not require complex data correction, data analysis or data interpretation. During the two-step acceptor photobleaching protocol, the FRET-induced changes in either fluorescence intensity or fluorescence lifetime are determined. A simple and helpful workflow for the analysis and interpretation of the results obtained during this two-step acceptor photobleaching protocol is then provided. Finally, by using a novel combination of FLIM- and intensity-based FRET data interpretation applied for the same cell, our method can be used to reliably distinguish trimeric from different types of dimeric (single-, double- or triple-dimeric) protein–protein interactions of three potential interaction partners in living cells.

## Materials and Methods

### Plasmids and Plasmid Construction

mTurquoise2-N1 (#54843, denoted as T) (Goedhart et al., 2012) and YPet-N1 (#54637, denoted as Y) (Nguyen and Daugherty, 2005) were purchased from addgene. pLego-mCherry (denoted as C) was kindly provided by Dr. Boris Fehse (Department of Stem Cell Transplantation, University Medical Center Hamburg-Eppendorf, Germany). FRET doublets (TY, TC, YC) and triplet (TCY, CTY, TYC) constructs were derived from pmVenus(L68V)-mTurquoise2 FRET positive control purchased from addgene (#60493) (Goedhart et al., 2012). YPet-mTurquoise2 (TY) and mCherry-mTurquoise2 (TC) were cloned by exchanging the sequence of mVenus in mVenus-L68V-mTurquoise2 to YPet and mCherry, respectively. YPet-mCherry (YC) was cloned from TY by exchanging the sequence of mTurquoise2 to mCherry. YPet-mCherry-mTurquoise2 (TCY) was obtained by inserting the linker sequence from mVenus-L68V-mTurquoise2 and mCherry into TY. mCherry-YPet-mTurquoise2 (TYC) was derived from TY by adding mCherry sequence and the linker sequence from mVenus-L68V-mTurquoise2 to the *N*-terminus of TY. YPet-mTurquoise2-mCherry (CTY) was obtained by inserting the linker sequence from mVenus-L68V-mTurquoise2 and the sequence of mTurquoise2 into YC. All cloning steps including sequence verification by Sanger sequencing were performed by Synbio Technologies (Monmouth Junction, United States). Plasmid DNA was transformed in Escherichia *coli* and purified using a GeneJet Mini Prep Kit (Thermo Fisher Scientific, Waltham, United States).

### Cell Culture

HEK 293T cell were seeded in T25 culture flasks (Sarstedt, Nümbrecht, United States) and cultured in DMEM high-glucose medium (Thermo Fisher Scientific, Waltham, United States) supplemented with 10% FCS (Pan-Biotech, Aidenbach, Germany), 1% GlutaMAX (Thermo Fisher Scientific, Waltham, United States) and 1% penicillin/streptomycin (Thermo Fisher Scientific, Waltham, United States) for up to five days at 5% CO_2_ and 37°C. For passaging, HEK 293T cells were washed in PBS (Thermo Fisher Scientific, Waltham, United States) and incubated in 1x trypsin/EDTA-Solution (Thermo Fisher Scientific, Waltham, United States) to dissolve cell contacts. To stop trypsin reaction, FCS-containing culture medium was added and cell density was assigned using a cell counting chamber (Marienfeld superior, Lauda-Königshofen, Germany). For imaging experiments, 10,000 cells per well were seeded in a 96-well glass bottom plate (Greiner, Kremsmünster, Austria). Cells were transfected with up to 0.2 μg/well plasmid DNA using TurboFect cell transfection reagent (Thermo Fisher Scientific, Waltham, United States) and used for imaging experiments the next day. For simultaneous transfection of two or three plasmids, one half or one third of the respective plasmid DNA was used to not exceed 0.2 μg total DNA/well.

### Confocal Laser Scanning Microscopy

Images for acceptor photobleaching experiments were acquired using a confocal laser scanning microscope (Nikon A1R) equipped with a 60× oil immersion objective (Nikon, plan apo lambda, N.A = 1.40), a PMT/GaAsP detector unit (Nikon, Tokio, Japan) and a cage incubator (Okolab, Ottaviano, Italy), the latter used to provide humidified and stable ambient conditions (37°C and 5% CO_2_). Fluorescence of mTurquoise2 was excited using the 405 nm laser line of a diode laser (Coherent, Santa Clara, United States) and fluorescence emission was detected in the spectral range of 465–500 nm. YPet was excited by the 514 nm or the 488 nm laser line of an argon laser (Melles Griot, Bensheim, Germany) and detected in the spectral range of 525–555 nm. The fluorescence of mCherry was excited by the 561 nm laser line of a solid-state laser (Coherent, Santa Clara, United States) and fluorescence emission was detected in the range of 570–620 nm. Laser power and detector gain were set such that about one third to a half of the detector’s dynamic range was used to allow for detection of fluorescence intensity increases without driving the detector into complete saturation.

### Fluorescence Lifetime Imaging (FLIM)

Fluorescence lifetime images were acquired using the FLIM upgrade kit (Picoquant, Berlin, Germany) for the Nikon 1AR confocal laser scanning microscope. Fluorescence of mTurquoise2 was excited using a pulsed laser source (PDL 828 Sepia II, Picoquant) at a wavelength of 444 nm and a repetition rate of 20 Mhz. Single photons and their arrival times were detected (PicoHarp300, 483/35 filter) using time correlated single photon counting (TCSPC) method. To avoid pile-up effects, excitation laser intensity was adjusted for each cell to keep maximum count rate below 2,000 kcps. Photons were counted for up to 30 cycles, for 2-step acceptor photobleaching experiments, cycle number was reduced to 6 cycles to reduce bleaching of YPet during image acquisition. Decay profiles were recorded using SymPhoTime 64 software (Picoquant, Berlin, Germany) and fitted by employing either two- or three-exponential reconvolution fits using a measured instrument response function (IRF). IRF was measured from fluorescein quenched with saturating concentrations of potassium iodide. For a comparative analysis, one-, two and three exponential tail fits or reconvolution fits using either a measured or a calculated IRF were applied. In fitting models employing tail fits, the fitting interval suggested by the software was not altered to allow comparability. For the display of the decay profiles, photon counts were normalized to the peak value. Peak value was set to time point 0 to better visualize the timescale of the fluorescence decay. FRET efficiencies were calculated from the lifetime of the donor-acceptor combination (τ_DA_) and the lifetime of the donor alone (τ_D_) using the following equation: E_FRET_ = (1 – (τ_DA_/τ_D_)).

### Two-Step Acceptor Photobleaching

Initial fluorescence intensities of mTurquoise2, YPet and mCherry or the initial lifetime of mTurquoise2 of the cells were documented by confocal laser scanning microscopy or FLIM, respectively. Thereafter, mCherry fluorescence was bleached by 561 nm laser at high laser power to about 5–10% of its initial intensity. Acquisition of confocal images or lifetime imaging was repeated using the same image setting as before. Accordingly, YPet fluorescence was bleached by 514 nm laser at high laser power below 5% of its initial intensity which was followed by a third acquisition of confocal images or lifetime images. For intensity-based measurements, the fluorescence intensity of the cell was subtracted by the background and normalized to the first image. For each bleaching step, FRET efficiencies were calculated from the fluorescence intensity of the FRET donor before (I_pre_) and after the photobleaching (I_post_) by the following formula: E_FRET_ = (1 – (I_pre_/I_post_)). For FLIM, the average amplitude weighted lifetime of the cell was measured. For each bleaching step, changes of the fluorescence lifetime were normalized to the lifetime of the previous image. In some experiments, instead of a two-step bleaching, a 1-step bleaching protocol using only one of the respective laser lines was applied.

### Statistics

For statistical analysis, data was tested by student’s *t*-tests or one-way ANOVA followed by a Tukey post hoc test using GraphPad Prism 5 (GraphPad Software, Inc., United States). Significance levels were *p* < 0.05 (^∗^), *p* < 0.01 (^∗∗^), *p* < 0.001 (^∗∗∗^), *p* < 0.0001 (^****^). In figures, error bars indicate standard error and n represents the number of measured cells.

## Results

### Basal Properties of the FRET Constructs

**FIGURE 1:**
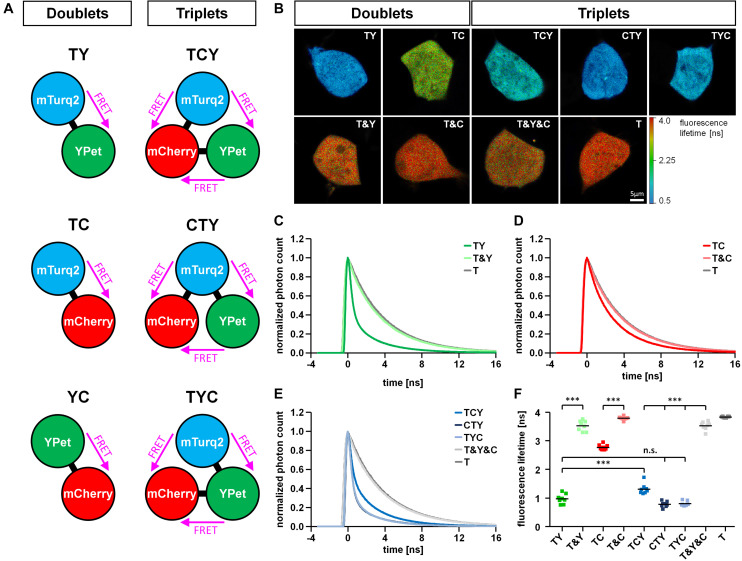
Basal properties of FRET triplet and doublet constructs. **(A)** Schematic drawing of FRET doublet and FRET triplet constructs composed of a combination of the fluorophores mTurquoise2 (T), YPet (Y) and mCherry (C). Note that within the triplet constructs, FRET can occur in three different directions. **(B)** Representative fluorescence lifetime images of FRET doublet (TY, TC) and FRET triplet constructs (TCY, CTY, TYC) expressed in HEK293T cells in comparison to the respective negative controls (T&Y, T&C, T&Y&C) and the FRET donor alone (T). **(C–E)** Average decay profiles of mTurquoise2 for different conditions (*n* = 8). The decay profile of the TY doublet shows a clear FRET-induced left shift compared to the negative control (T&Y) and the donor alone (T) **(C)**. The left shift observed in the TC doublet was less prominent **(D)**. FRET-induced left shifts of the fluorescence decay observed in the FRET triplets was different depending on the position of T, Y and C within the linear construct **(E)**. **(F)** Average amplitude weighted lifetimes of all FRET constructs, their respective negative controls and the FRET donor mTurquoise2 alone (*n* = 8). Note the differences in lifetime reduction according to the type of interacting fluorophores in the FRET doublets (TY, TC) or the positioning of the fluorophores within the FRET triplet constructs (TCY, CTY, TYC).

We designed FRET constructs composed of blue-fluorescent mTurquoise2 (T), yellow-fluorescent YPet (Y) and red-fluorescent mCherry (C) in different compositions (Figure 1A). These fluorophores were chosen for the following reasons: 1) The spectral properties of these fluorophores exhibit a clear overlap between the emission spectrum of the FRET donor and the excitation spectrum of the FRET acceptor for every possible interaction (TY, TC, and YC) (Supplementary Figures 1A–C), which is a prerequisite for the final energy transfer. 2) The fluorophores can be excited and detected specifically with common equipment for confocal laser scanning microcopy. 3) The photostability of the fluorophores is high enough to avoid bleaching due to image acquisition (Supplementary Figure 2C), which has been problematic especially for red fluorophores like mRuby3 used in preliminary experiments. 4) For fluorescence lifetime imaging, mTurquoise2 has shown a favorable mono-exponential decay (Goedhart et al., 2012) and good FRET efficiencies are obtained for both mTurquoise2 and YPet, with YPet representing an optimized yellow FRET acceptor variant for cyan donors (Nguyen and Daugherty, 2005). For our FRET constructs, we connected the fluorophores with a short linker (SGLRSSDPPVAT) that was shown to enable high FRET efficiencies between coupled fluorophores (Mastop et al., 2017). We arranged the fluorophores in a way to obtain either the doublet constructs TY, TC and YC or the triplet constructs TCY, CTY, TYC (Figure 1A). The doublet constructs only have one possible direction of FRET (representing a dimeric interaction), the triplet constructs can account for three possible directions of FRET (representing a trimeric interaction). Therefore, we varied the positions of the fluorophores in the FRET triplet constructs to cover different distances within the linear chain of the protein which might lead to differences in observed FRET efficiencies. Next, we transfected our FRET doublet constructs into HEK293T cells and examined the changes in fluorescence intensity with acceptor photobleaching experiments of TY, TC and YC doublets and their respective controls (Supplementary Figure 2A). The highest unquenching of donor intensity after photobleaching of the acceptor could be observed for the TY construct, while unquenching of the TC and YC constructs remained rather low, indicating that the FRET efficiency is highest in the TY construct. We calculated absolute FRET efficiencies from the acceptor photobleaching experiments (TY: 71.2 ± 1.5%, TC: 29.1 ± 0.7%, YC: 28.6 ± 2.0%; Supplementary Figure 2B). To further investigate the FRET in our triplet and doublet constructs, we analyzed the fluorescence lifetime (τ) by fluorescence lifetime imaging microscopy (FLIM). With our experimental configuration we were able to detect the lifetime of mTurquoise2 giving us the opportunity to study TY- and TC-related but not YC-related FRET. For initial experiments, we used the mTurquoise2-YPet FRET doublet (TY) in comparison to the FRET donor alone (mTurquoise2) to determine the best fitting parameters for our FLIM data. We tested the performance of a tail fit versus a reconvolution fit using either a measured or a calculated instrument response function (IRF displayed in Supplementary Figure 3) for one-, two- and three-exponential decay models (Supplementary Figure 4). For the fluorescence decay of the donor unaffected by FRET (donor only), we observed a short lifetime component directly after the beginning of the decay contributing ∼10% to the total decay amplitude. We therefore decided to fit the data with a 2-exponential model, although mTurquoise2 has been reported to show mono-exponential decay (Goedhart et al., 2012). In contrast, the FRET-affected donor lifetime showed a 3-exponential decay, so we decided to fit our FRET samples with this model. The first lifetime component in this 3-exponential decay was very short (t_1_ ∼ 0.5 ns), but accounted for ∼66% of the total decay amplitude. Therefore, the tail fit, whose adaptation interval starts shortly after the onset of fluorescence decay, could not optimally cover this part of the decay, resulting in a longer average donor lifetime at occurrence of FRET compared to the reconvolution fit (Supplementary Figures 4D,E). However, for samples unaffected by FRET, tail fit and reconvolution fit performed almost equally well. Considering that by definition the lifetime τ corresponds to the time needed to decay to 1/e of the initial fluorescence intensity, the reconvolution fit and the average amplitude weighted lifetime was matching best the observed fluorescence decays especially for situations in which high FRET occurred. In addition, the calculated FRET efficiencies of the TY construct obtained from the amplitude-weighted lifetime (74.6 ± 1.6%) corresponded better to the FRET efficiencies observed by acceptor photobleaching experiments (Supplementary Figure 2B) than the FRET efficiencies calculated from the intensity-weighted lifetime (40.7 ± 2.7%).

After identifying the best fitting parameters, we performed a comparative analysis of the FRET doublet and triplet constructs. The reduction of the fluorescence lifetime due to FRET was compared to the lifetime of the negative controls, which consisted of a combination of the separated fluorophores (Figures 1B–F). In the T&Y, T&C and T&Y&C combinations, the lifetime of mTurquoise2 was comparable to the lifetime of mTurquoise2 alone (T) with a slight reduction in T&Y and T&Y&C transfected cells most likely due to the non-specific FRET observed between T&Y (Supplementary Figure 2B). However, for the FRET doublet constructs, the donor lifetime was significantly reduced to 0.97 ± 0.06 ns for TY and 2.77 ± 0.03 ns for TC. The observation of the much stronger reduction in fluorescence lifetime for the TY compared to the TC construct are in line with the acceptor photobleaching data (Supplementary Figures 2A,B) indicating that FRET in the TY construct is much more efficient than in the TC doublet. The effect of FRET can also be seen by a clear left shift of the fluorescence decay which is more pronounced for TY than for TC (Figures 1C,D). For the FRET triplet constructs, there are significant differences in fluorescence lifetime of mTurquoise2 depending on the position of the fluorophores within the protein chain. In the TCY construct, the TY-FRET pair, which usually gives rise to the highest FRET is separated by mCherry. Therefore, lifetime shortening of mTurquoise2 is not as pronounced as with the CTY and TYC constructs, where the TY-FRET pair is closer together. Donor lifetime reduction in the TCY construct is also less pronounced than for the TY-doublet, which indicates a dominant role of the TY-FRET for the overall FRET efficiency of the triple constructs. Nevertheless, the lifetime of mTurquoise2 is slightly, but not significantly more reduced in CTY and TYC constructs than in the TY-doublet, indicating that the TC-FRET contributes to a further reduction of lifetime here.

### Differentiation Between Dimeric and Trimeric Interactions

**FIGURE 2:**
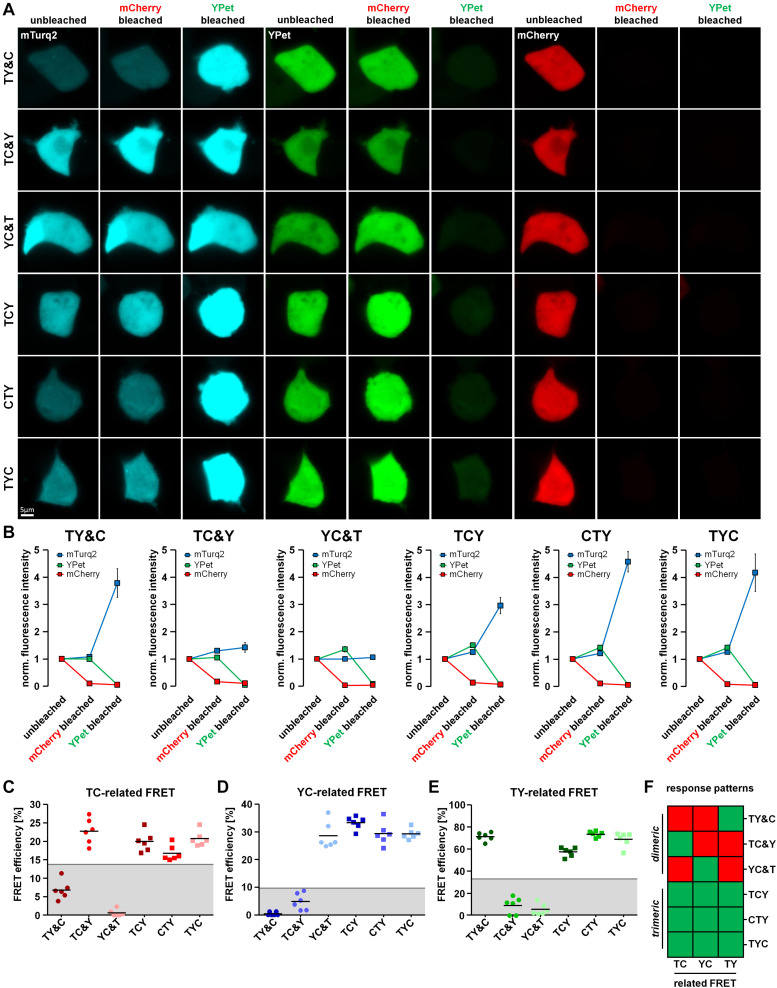
Differentiation of dimeric and trimeric interactions using fluorescence intensity-based measurements. **(A)** HEK293T cells transfected with either the FRET triplet constructs (TCY, CTY, TYC) or the FRET doublets with their respective missing fluorophore (TY&C, TC&Y, YC&T). Representative images show the fluorescence intensity of mTurquoise2, YPet and mCherry before bleaching (unbleached), after the first bleaching step (mCherry bleached) and after the second bleaching step (YPet bleached). **(B)** Average fluorescence intensity of mTurquoise2 (mTurq2; blue), YPet (green) and mCherry (red) normalized to the first image acquired (*n* = 6). After mCherry bleaching, the YPet fluorescence intensity increased in YC&T, TCY, CTY and TYC and the mTurquoise2 fluorescence intensity increased in TC&Y, TCY, CTY and TYC, only. There was no increase in YPet fluorescence intensity in TY&C and TC&Y samples and no increase in mTurquoise2 fluorescence intensity in TY&C and YC&T samples due to the absence of specific YC- and TC-FRET. After YPet bleaching, the mTurquoise2 fluorescence intensity drastically increased in TY&C, TCY, CTY and TYC, whereas the mTurquoise2 fluorescence intensity remained rather constant in TC&Y and YC&T samples due to the absence of specific TY-FRET. The fluorescence increase corresponded to the unquenching of the FRET donor fluorescence intensity after bleaching its respective FRET acceptor, thereby abolishing FRET between them. **(C–E)** FRET efficiencies calculated from the change in fluorescence intensity of mTurquoise2 [TC-related FRET, **(C)**] and YPet [YC-related FRET, **(D)**] after mCherry bleaching and the FRET efficiency obtained from the change of mTurquoise2 fluorescence after YPet bleaching [TY-related FRET, **(E)**] (*n* = 6). Threshold values set to discriminate interaction-specific FRET from non-specific FRET are indicated by gray lines. Note the different response patterns of FRET doublets compared to those from FRET triplets. **(F)** Color coded response pattern of fluorescence intensity-based measurements. A Yes-response (occurrence of specific FRET) is indicated by a green and a No-response (no or non-specific FRET) is indicated by a red square. According to these patterns, dimeric interactions (FRET doublets) can be clearly discriminated from trimeric interactions (FRET triplets).

The formation of dimeric and trimeric protein complexes is a well-known phenomenon in cellular signal transduction and homeostasis. As a model for the FRET-based detection of dimeric and trimeric protein interactions, we designed FRET doublet and FRET triplet constructs, respectively. We intended to distinguish such interactions by exploiting the unique properties of FRET doublets as compared to FRET triplets within a 3-fluorophore setup. For this purpose, we established a two-step acceptor photobleaching protocol to allow the separate analysis of mCherry and YPet-related FRET fractions in living cells. We transfected TCY, TYC and CYT FRET constructs as well as the FRET doublets together with the respective missing fluorophore (TY&C, TC&Y, YC&T) into HEK293T cells. Thereafter, we first bleached mCherry and analyzed the changes in fluorescence intensity of YPet and mTurquoise2 (Figures 2A,B). The intensity of YPet was moderately unquenched in YC&T, TCY, CTY and TYC expressing cells, indicating, as expected, the presence of a rather low-efficient YC-FRET (please also see Supplementary Figures 2A,B). However, in the TY&C and TC&Y conditions, unquenching of YPet after mCherry bleaching was negligible, suggesting the absence of YC-FRET in these cells. Likewise, the intensity of mTurquoise2 was only moderately increased in TC&Y, TCY, CTY and TYC conditions, whereas in TY&C and YC&T, unquenching of mTurquoise2 was absent (Figures 2A,B). In the second step of the experimental protocol, we bleached YPet and subsequently analyzed the unquenching of mTurquoise2. In TY&C, TCY, CTY and TYC expressing cells, we detected a strong increase of mTurquoise2 fluorescence intensity, while in TC&Y and YC&T expressing cells, only a minor increase due to non-specific FRET between Y&T was observed (Figures 2A,B). This non-specific increase of mTurquoise2 fluorescence in TC&Y and YC&T was not related to photoconversion of YPet or other changes in background fluorescence induced by the bleaching protocol as indicated by control experiments bleaching YPet in the absence of mTurquoise2 (Supplementary Figure 2D). As far as the triplet constructs are concerned, mTurquoise2 unquenching was lowest in TCY where the strong TY-FRET pair is separated by mCherry. These observations are in line with the reduction in fluorescence lifetime observed for the triplet FRET constructs in previous experiments (Figure 1F). From these data, we were able to deduce unique response patterns of FRET doublets and triplets, respectively, which allowed us to determine the number of interacting partners in this 3-fluorophore set up. These patterns are described later in section “Interpretation of the Response Patterns of the Two-Step Acceptor Photobleaching Protocol.”

**FIGURE 3:**
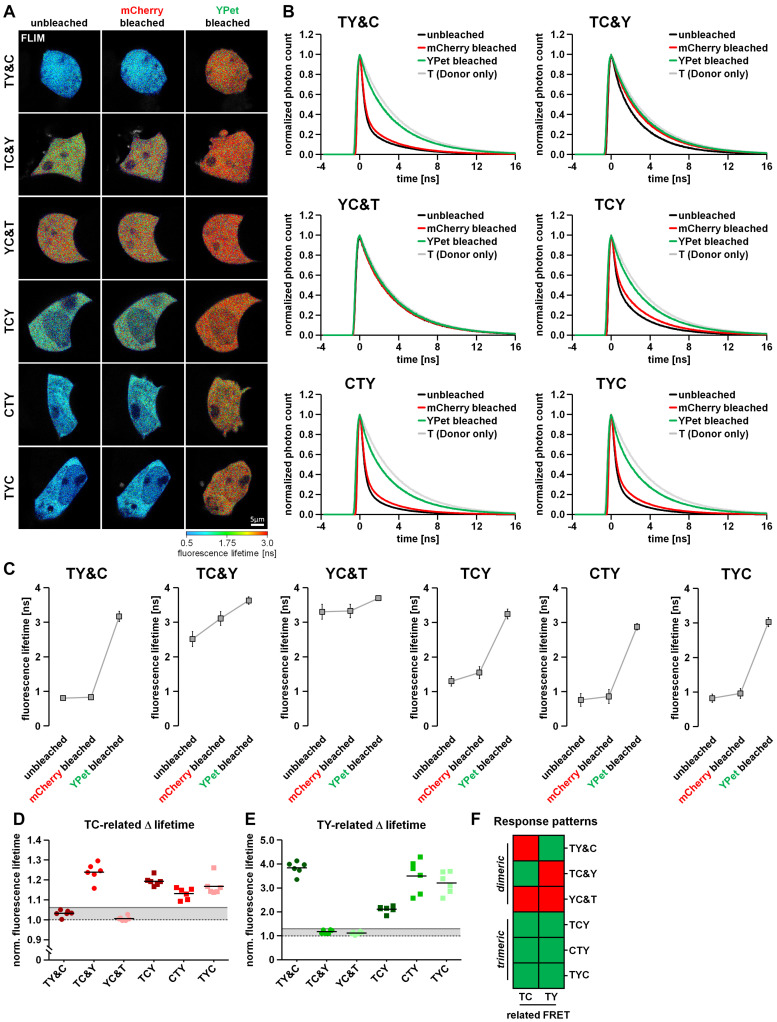
Differentiation of dimeric and trimeric interactions using fluorescence lifetime-based measurements. **(A)** HEK293T cells transfected with either the FRET triplet constructs (TCY, CTY, TYC) or the FRET doublets with their respective missing fluorophore (TY&C, TC&Y, YC&T). Representative images show the color-coded fluorescence lifetime of mTurquoise2 before bleaching (unbleached), after the first bleaching step (mCherry bleached) and after the second bleaching step (YPet bleached). **(B)** Average profiles of mTurquoise2 fluorescence decay (*n* = 6). Note the sequential reversion of the FRET-induced left shift of mTurquoise2 fluorescence decay after the first and the second bleaching step. **(C)** Change in average amplitude weighted lifetime of the cells during the two-step bleaching protocol (*n* = 6). After mCherry bleaching, the fluorescence lifetime increased moderately in TC&Y and slightly in TCY, CTY and TYC samples, whereas it remained rather constant in TY&C and YC&T samples. In contrast, after YPet bleaching, the fluorescence lifetime increased drastically in TY&C, TCY, CTY and TYC, whereas in TC&Y and YC&T samples only a moderate increase was observed. Note the different basal fluorescence lifetime depending on the combination and composition of the FRET constructs. **(D,E)** Relative change in fluorescence lifetime of mTurquoise2 after mCherry bleaching [TC-related Δlifetime, **(D)**] and the additional relative change in fluorescence lifetime after YPet bleaching [TY-related Δlifetime, **(E)**] (*n* = 6). Gray lines indicate threshold values to discriminate FRET-specific from non-specific lifetime changes of the donor. **(F)** Color coded response pattern of fluorescence lifetime-based measurements. For each matrix, a Yes-response (occurrence of specific FRET) is indicated by a green and a No-response (no or non-specific FRET) is indicated by a red square. According to these patterns, dimeric interactions (FRET doublets) can be clearly discriminated from trimeric interactions (FRET triplets).

As an additional detection method for FRET we used the previously described two-step acceptor photobleaching protocol and determined subsequent TY-FRET- and TC-FRET-related changes in mTurquoise2 fluorescence lifetime using FLIM. For this purpose, we transfected HEK293T cells with our FRET triplet constructs (TCY, TYC and CYT) as well as the FRET doublets together with the respective missing fluorophore (TY&C, TC&Y, YC&T). After mCherry bleaching, fluorescence lifetime of mTurquoise2 increased in TC&Y-, TCY-, CTY- and TYC-expressing cells, indicating the occurrence of FRET between mTurquoise2 and mCherry under these conditions (Figures 3A,C). Compared to TC&Y, the change in fluorescence lifetime of the FRET triplet constructs was rather small, probably due to simultaneously occurring strong TY-FRET in these constructs which has a dominant influence on the lifetime of mTurquoise2 and was not affected by this bleaching step. In contrast, donor lifetime in TY&C- and YC&T-expressing cells remained nearly constant, indicating that under these conditions no FRET occurred between mTurquoise2 and mCherry. After YPet bleaching, the fluorescence lifetime of mTurquoise2 in TY& C-, TCY-, CTY- and TYC-expressing cells increased strongly, which underlines the significant influence of TY-associated FRET in these constructs. In TC&Y and YC&T expressing cells, however, an unexpected moderate increase in donor lifetime was observed, most likely due to the relatively strong non-specific FRET between T and Y as separate fluorophores (Figures 3C and Supplementary Figures 2A,B). After the last bleaching step, the fluorescence lifetime remained below the lifetime of the mTurquoise2 donor alone (∼3.9 ns, compare T in Figure 1F) in all condition, indicating the presence of residual FRET due to incomplete acceptor photobleaching. The effect of FRET reduction after each bleaching step is also apparent from the profiles of fluorescence decay (Figure 3B).

### Interpretation of the Response Patterns of the Two-Step Acceptor Photobleaching Protocol

Using the two-step acceptor photobleaching protocol and intensity- as well as fluorescence lifetime-based FRET analyses, we obtained conclusive response patterns for the various doublet and triplet combinations transfected in HEK293T cells. These patterns can be used to discriminate dimeric from trimeric interactions. To facilitate systematic analysis and evaluation of the results obtained, we organized the data in a visual decision matrix. For this, we plotted the TC- and YC-related FRET efficiencies derived from the change in fluorescence intensity of mTurquoise2 and YPet after mCherry bleaching (Figures 2C,D) as well as the TY-related FRET efficiency derived from the change in fluorescence intensity of mTurquoise2 after YPet bleaching (Figure 2E). To distinguish between specific and non-specific FRET after acceptor photobleaching (the latter being FRET between separate fluorophores; Supplementary Figure 2A), we established threshold values for the FRET efficiency of each fluorophore combination on the basis of the non-specific relative fluorescence increase plus five times its standard deviation (TC-FRET = 13.8%, YC-FRET = 9.8%, TY-FRET = 33.1%, gray lines, Figures 2C–E). Using these thresholds, we obtained an unambiguous response pattern of donor fluorescence intensity for each FRET-pair during the 2-step acceptor photobleaching protocol. The resulting decision matrix (Figure 2F) helps to discriminate dimeric (TY&C, TC&Y, YC&T) from trimeric protein–protein interactions (TCY, CTY and TYC). In a similar fashion, we plotted relative changes in fluorescence lifetime of mTurquoise2 after performing the acceptor photobleaching protocol (Figures 3D,E). As thresholds, we used the ratio of fluorescence lifetime of mTurquoise2 alone to lifetime of the combined but separated fluorophores (please see T&Y, T&C and T in Figure 1F) plus five times the standard deviation (TC = 1.06, TY = 1.32, gray lines, Figures 3D,E). Importantly, the FLIM-FRET pattern of the TY- and TC-related FRET closely resembled the pattern of the TY- and TC-related FRET observed in intensity-based analyses (Figures 2C,E, 3D,E). As a result, we obtained a second decision matrix, that is also able to distinguish dimeric from trimeric protein–protein interactions (Figure 3F), although YC-related effects could not be assessed with our FLIM configuration.

### Discrimination of Different Types of Dimeric Interactions

**FIGURE 4:**
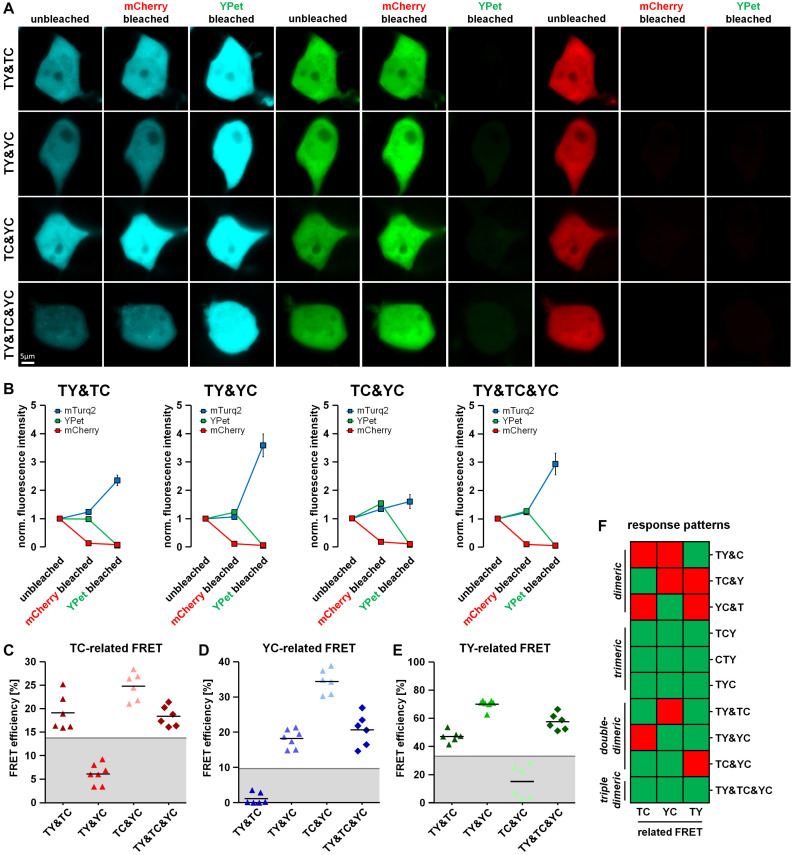
Discrimination of different types of dimeric interactions among three potential interaction partners using fluorescence intensity-based measurements. **(A)** HEK293T cells were co-transfected with two doublet constructs (TY&TC, TY&YC, TC&YC) or a combination of all three FRET doublets (TY&TC&YC). Representative images showing the fluorescence intensity of mTurquoise2, YPet and mCherry before bleaching (unbleached), after the first bleaching step (mCherry bleached) and after the second bleaching step (YPet bleached). **(B)** Average fluorescence intensity of mTurquoise2 (blue), YPet (green), and mCherry (red) normalized to the first image acquired (*n* = 6). After mCherry bleaching, the YPet fluorescence intensity increased in TY&YC, TC&YC and TY&TC&YC samples, while it slightly decreased in TY&TC. In contrast, the intensity of mTurquoise2 increased in TY&TC, TC&YC and TY&TC&YC but remained rather constant in TY&YC samples. After YPet bleaching, the mTurquoise2 fluorescence intensity drastically increased in TY&TC, TY&YC and TY&TC&YC, only. **(C–E)** FRET efficiencies calculated from the change in fluorescence intensity of mTurquoise2 **(C)** and YPet **(D)** after mCherry bleaching (first bleaching step) and the from the additional relative change of mTurquoise2 fluorescence after YPet bleaching [**(E)**, second bleaching step] for double transfections TY&TC, TY&YC and TC&YC as well as the triple transfection TY&TC&YC (*n* = 6). Threshold values to discriminate interaction-specific FRET from non-specific FRET are indicated by gray lines. **(F)** Updated color-coded response patterns and decision matrix of fluorescence intensity-based measurements. For each direction of FRET, a Yes-response (occurrence of specific FRET) is indicated by a green and a No-response (no or non-specific FRET) is indicated by a red square. The patterns can be used to discriminate dimeric from trimeric and double-dimeric interactions. However, the response patterns are not suitable to distinguish between trimeric interactions and triple-dimeric interactions in our experimental approach.

**FIGURE 5:**
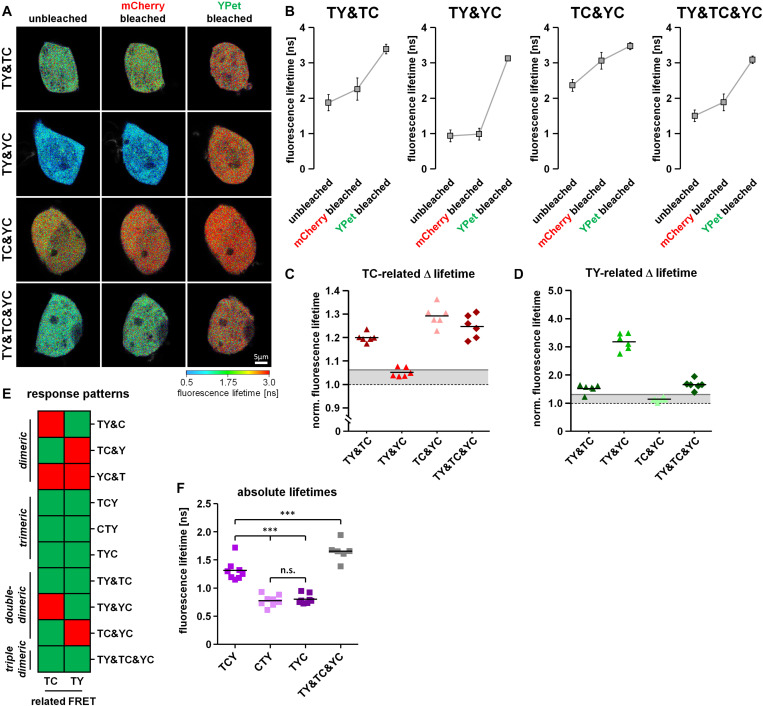
Discrimination of different types of dimeric interactions among three potential interaction partners using fluorescence lifetime-based measurements. **(A)** Representative images showing the color-coded fluorescence lifetime of mTurquoise2 before bleaching (unbleached), after the first bleaching step (mCherry bleached) and after the second bleaching step (YPet bleached). **(B)** Change in average amplitude weighted lifetime of the cells during the two-step bleaching protocol (*n* = 6). After mCherry bleaching, the fluorescence lifetime increased in TY&TC, TC&YC and TY&TC&YC samples, but remained constant in TY&YC. After YPet bleaching, the fluorescence lifetime strongly increased in TY&TC, TY&YC and TY&TC&YC, but only moderately in TC&YC. **(C,D)** Relative change in fluorescence lifetime of mTurquoise2 after mCherry bleaching [**(C)**, first bleaching step] and the additional relative change in fluorescence lifetime after YPet bleaching [**(D)**, second bleaching step] (*n* = 6). Gray lines indicate threshold values to discriminate FRET-specific from non-specific lifetime changes of the donor. **(E)** Updated color-coded response patterns and decision matrix of fluorescence lifetime-based measurements. For each direction of FRET, a Yes-response (occurrence of specific FRET) is indicated by a green and a No-response (no or non-specific FRET) is indicated by a red square. Without information about YC-related FRET, these patterns cannot be used to discriminate dimeric from trimeric and double-dimeric interactions as it was shown for the intensity-based patterns. **(F)** However, the different basal fluorescence lifetimes of the combination of all three dimers versus each of the FRET triplet constructs allow for their discrimination of multi-dimeric and trimeric interactions which was not possible from the intensity-based pattern.

Proteins often form dimers with more than one interaction partner. A difficulty in the analysis of 3-fluorophore FRET approaches is therefore to distinguish trimeric protein–protein interaction states from simultaneously occurring but varying dimeric protein–protein interactions of the same proteins. To address this question, we co-transfected HEK293T cells with a combination of two different FRET doublets (TY&TC, TY&YC, TC&YC) or a combination of all FRET doublets (TY&TC&YC). Using our acceptor photobleaching protocol, we were able to detect unquenching of the mTurquoise2 fluorescence after mCherry bleaching in TY& TC-, TC&YC- and TY&TC&YC- but not in TY&YC-transfected cells (Figures 4A,B), a phenomenon that can be attributed to the presence or absence of the TC doublet. Moreover, YPet unquenching after mCherry bleaching was observed in TC&YC-, TY&YC- as well as TY&TC&YC- but not in TY&TC-transfected cells due to the presence or absence of the YC doublet. Lastly, unquenching of mTurquoise2 after YPet bleaching was detected in TY&TC-, TY&YC- and TY&TC&YC- but not in TC&YC-expressing cells. Similarly, we analyzed the change in fluorescence lifetime of mTurquoise2 after mCherry and YPet bleaching (Figures 5A,B). A corresponding significant increase in mTurquoise2 lifetime was observed after mCherry bleaching in TY&TC-, TC&YC- and TY&TC&YC-expressing cells and after YPet bleaching in TY&TC-, TY&YC- and TY&TC&YC-expressing cells. To facilitate systematic analysis and evaluation of the results obtained, we again organized the data in our visual decision matrix, thereby plotting the FRET efficiencies of the TC-, YC- and TY- related FRET (Figures 4C–E). We again used the established threshold values for each fluorophore combination to distinguish between specific and non-specific FRET (gray lines, compare Figures 2C–E). For the TC-related FRET, only TY&TC-, TC&YC- and TY&TC&YC-expressing cells showed an increase above the threshold due to the presence of the TC-FRET pair in these conditions. The YC-related FRET was only above the threshold for TY&YC-, TC&YC- and TY&TC&YC-transfected cells, a phenomenon caused by the YC-FRET pair in these combinations. Finally, for the TY-related FRET there was an additional increase above the threshold in TY&TC-, TY&YC- and TY&TC&YC-expressing cells. We added the response patterns of the changes in fluorescence intensity to the previous graph (Figure 4F) showing now all interactions that were investigated. On the basis of these patterns it is possible to discriminate single-dimeric from trimeric as well as trimeric from double-dimeric interactions. Unfortunately, however, this method cannot distinguish between triple-dimeric and trimeric protein–protein interactions. Similarly, we plotted the relative change in mTurquoise2 fluorescence lifetime in response to acceptor photobleaching of mCherry and YPet (Figures 5C,D). The already established thresholds for differentiating between specific FLIM-FRET changes and non-specific FLIM-FRET changes were kept constant (Figures 3D,E). We obtained similar results, showing a TC-FRET-related increase in fluorescence lifetime in TY&TC-, TC&YC- and TY&TC&YC-transfected cells and a TY-FRET-related increase in fluorescence lifetime in TY&TC-, TY&YC- and TY&TC&YC-expressing cells. The updated decision matrix indicates that the present setup is not sufficient to distinguish FRET patterns obtained from TY&TC- and FRET triplet-expressing cells (Figure 5E). Also, a FLIM-FRET-based differentiation of multi-doublet (TY&TC&YC)-expressing cells from FRET triplet-expressing cells is not possible. But importantly, compared to the FRET triplets, the fluorescence lifetime of mTurquoise2 obtained in the multi-doublet combination is increased due to dilution of highly efficient TY-FRET with low-efficient TC-FRET in this combination (Figure 5F).

### Correlation of Fluorescence Lifetime Values With FRET-Efficiencies Obtained by Acceptor Photobleaching

**FIGURE 6:**
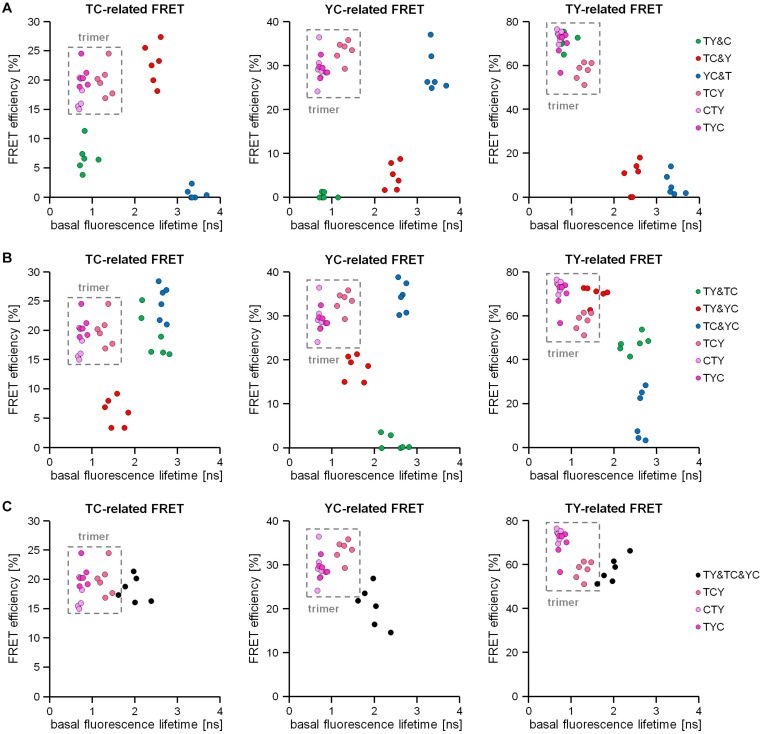
Discrimination of trimeric from dimeric interactions by using a combination of acceptor photobleaching and the basal donor fluorescence lifetime assessment. Correlation of the basal fluorescence lifetime of mTurquoise2 with the intensity-based TC-FRET, YC-FRET and TY-FRET-related FRET efficiencies obtained from the acceptor photobleaching of the same cell (*n* = 6 each). **(A)** Discrimination of trimeric interactions (FRET triplets TCY, CTY, TYC) from dimeric interactions (FRET doublets TY&C, TC&Y, YC&T) in this correlation. In all three FRET types (T > Y; T > C; Y > C) investigated, the trimeric interaction-related data points occupy the upper left quadrant of the correlation plots (marked as a gray rectangles), while the data points of the dimeric interactions are predominantly located in other quadrants. **(B)** Discrimination of the trimeric interactions (FRET triplets TCY, CTY, TYC) from double-dimeric interactions (double FRET doublets TY&TC, TY&YC, TC&YC) using the same correlation plot system. Again, data points derived from double-dimeric interactions are mostly located outside the upper left quadrant. **(C)** Correlation plots deduced from trimeric interactions (FRET triplets TCY, CTY, TYC) and triple-dimeric interactions (TY&TC&YC). Note that due to dilution effects on mTurquoise2 lifetime in the basal unbleached state as well as distinguishable responses after acceptor photobleaching, data distribution in TY&TC&YC samples clearly differs from data distribution in TCY, CTY or TYC samples, respectively.

As in section “Discrimination of Different Types of Dimeric Interactions,” deduced response patterns from the 2-step acceptor photobleaching protocol can help to distinguish between simple or double-dimeric from trimeric interactions, but cannot provide sufficient information to distinguish triple-dimeric interactions from trimeric interactions. To overcome this problem, we intended to combine information from the mTurquoise2-related fluorescence lifetime measurements with information obtained from the FRET efficiencies detected during two-step bleaching measurements. For this, we first transfected HEK293T cells with our FRET doublets or FRET triplets and measured the basal, unbleached fluorescence lifetime. Thereafter, we applied our 2-step acceptor photobleaching protocol to measure TC-, YC- or TY-FRET related FRET efficiencies present in the cytosol of same cell. We plotted a correlation of this information and observed that the data points for the FRET triplets were ordered always on the upper left part of the plot since they show the strongest lifetime reductions and high FRET efficiencies for all FRET pairs, respectively (Figure 6A). In contrast, FRET doublets were often placed outside this area with the exception of the TY doublet, which showed an overlap with the trimer-associated pattern with regard to TY-FRET. We concluded that trimeric interactions can be identified (and reliably distinguished from dimeric interactions) if the collected data points are consistently positioned in the upper left quadrant of the aforementioned correlation plot. Next, we analyzed whether this correlation procedure also allows us to distinguish double-dimeric interactions from trimeric interactions (Figure 6B). Again, also in these experiments, the data points for TY&TC-, TY&YC- and TC&YC-expressing cells could be distinguished from data points of FRET triplet-expressing cells by a different position in the correlation plot. Finally, we used this method to investigate our triple-dimeric combination TY&TC&YC, which had been undistinguishable from the FRET triplets in our previous experiments (see Figures 4F, 5E). Although the values for the triple-dimeric combination are closer to the position of the FRET-triplets in the correlation plot, the triple-dimeric combination shows higher average donor lifetime values and reduced FRET efficiencies especially for the YC-related FRET (Figure 6C). This is most likely caused by diluting TC and YC-related FRET effects, which are absent in trimeric interactions.

## Discussion

In the present manuscript, we report a method for the discrimination of dimeric and trimeric protein–protein interactions using a three-fluorophore setup and a common confocal microscopy configuration. We established a two-step acceptor photobleaching protocol and subsequently investigated FRET response patterns with intensity-based methods as well as FLIM in living cells, expressing either dimeric, trimeric or multiple dimeric FRET constructs consisting of mTurquoise2, YPet and/or mCherry. Our method also provides guidance for the interpretation of the responses in fluorescence intensity and fluorescence lifetime obtained after each bleaching step by organizing the data into comprehensive decision matrices and correlation plots that help to distinguish between dimeric and trimeric interaction states of the above mentioned fluorescent proteins. This method can be successfully applied to fluorescently labeled proteins of interest to study the occurrence of trimeric protein–protein interactions in living cells. Importantly, by combining FLIM- and intensity-based FRET data acquisition and interpretation, our method allows to distinguish trimeric from different types of dimeric (single-, double- or triple-dimeric) protein–protein interactions of three potential interaction partners in the physiological setting of living cells. In this context, special attention was paid to distinguish triple-dimeric interactions from trimeric interactions of three potential interaction partners, which in our hands is not possible with solely intensity-based or solely fluorescence lifetime-based methods. This limitation, however, may also apply to studies investigating trimeric protein–protein interactions by analyzing FRET-induced sensitized emission of the acceptor (Sun et al., 2010; Hoppe et al., 2013; Scott and Hoppe, 2016). However, by combining FRET-efficiencies derived from intensity-based acceptor photobleaching experiments with the basal, unbleached fluorescence lifetime of the donor, we were able to discriminate reliably trimeric from triple-dimeric interactions. Nevertheless, the discrimination of these types of interactions must be performed with great care and a sufficient number of replicates as well as independent experiments, so that an adequately conclusive correlation plot between the basal donor lifetime and the FRET efficiencies derived from acceptor photobleaching can be obtained. In the biological context, proteins involved in the formation of heterotrimers can also exist as a mixture of heterotrimers, heterodimers and the respective missing monomers, or exclusively as independent monomers. Results of a simulation we performed, representing the application of our experimental approach to such a situation, indicate that these states can also be distinguished in principle by our method (Supplementary Data Sheet 4). However, these simulations should be verified experimentally in further future analyses.

Due to cell movement and fluorescent protein diffusion during relatively long bleaching intervals, researchers are often reluctant to use acceptor photobleaching techniques to study protein–protein interactions in living cells. One reason for that is that a pixel-accurate localization of FRET within the image is limited to fixed samples, in which pre- and post-bleach images can be precisely assigned to each other. Its accuracy in localizing FRET in living cells and its suitability for time-lapse-imaging are the major advantages of corrected FRET and spectral unmixing approaches over acceptor photobleaching. In our methodological approach, we have abstained from pixel-based accuracy in favor of performing our measurements in living cells. By bleaching and measuring the average fluorescence intensity of the whole cell, however, we were able to perform reliable FRET measurements, because all molecules contributing to FRET were similarly affected and detected. FRET efficiencies that we calculated from acceptor photobleaching experiments using our FRET doublets (TY, TC and YC; Supplementary Figure 2B) were very similar to the FRET efficiencies obtained in other studies that used similar methodology as well as the same fluorophores [for TY, TC, YC FRET efficiency please see (Scott and Hoppe, 2015), for TC lifetime and FRET efficiency additionally see (Mastop et al., 2017)]. With respect to the relatively high FRET efficiency of the TY construct, that was also observed by Scott and Hoppe (2015) using acceptor photobleaching measurements, it has been shown that the FRET efficiencies of dimers consisting of mTurquoise2 and various yellow fluorescent protein variants can behave differently even though the latter variants are closely related. Indeed, combinations of mTurquoise2 (or other cyan protein variants) with further yellow fluorophores may yield significantly lower FRET efficiencies (Koushik et al., 2006; Vogel et al., 2006; Scott and Hoppe, 2015; Mastop et al., 2017). The reasons for these differences are unclear. Nonetheless, a potential impact of fluorescence protein heterodimerization on FRET efficiency has been discussed (Kotera et al., 2010; Pope et al., 2020). In addition, the considerable FRET efficiency of mTurquoise2-YPet constructs may be due to the high extinction coefficient of YPet compared to other yellow fluorescent proteins, which increases its Förster radius to mTurquoise2 (Scott and Hoppe, 2015). Moreover, higher FRET values than those expected according to the Förster theory may be attributed to further interactions of the fluorescence protein dimers, e.g., the heterodimerization mentioned earlier, which may change either the spacing or the orientation of the fluorophores and thus increase the orientation factor κ^2^ beyond the assumed value of 2/3 (Scott and Hoppe, 2015). In this context, other authors also discuss that such a prediction *per se* may not be accurate for fluorescent proteins because they do not undergo much rotational diffusion during the short, excited state due to their relatively large molecular weight (Bajar et al., 2016).

In addition to the fluorescence intensity-based measurement, we used FLIM to detect different responses to acceptor photobleaching. Due to technical limitations of our FLIM setup, we were able to detect solely the lifetime of mTurquoise2, thereby not allowing us to study YC-FLIM-FRET-related changes. Therefore, in contrast to our intensity-based FRET data, our FLIM-based FRET data could not be used alone to distinguish trimeric from double-dimeric protein–protein interactions. This limitation can be overcome if FLIM systems are used that are also capable of analyzing YPet lifetime in living cells. In our analyses, the lifetime of mTurquoise2 alone was similar to the lifetime reported in other studies (Goedhart et al., 2012; Martin et al., 2018). The presence of a strong FRET had a major influence on the decay profile of mTurquoise2 by switching it from mono-exponential to multi-exponential decay. This effect of FRET on the exponentiality of fluorescence decay has already been reported for other fluorophores and is thought to be caused by subpopulations of differently efficient FRET pairs within a sample (Padilla-Parra et al., 2009; Godet and Mély, 2019). The best fit for a FRET-influenced mTurquoise2 decay profile was achieved by a 3-exponential reconvolution fit when reading out of the amplitude weighted lifetime, which in our opinion best represented the observed fluorescence decay and resulting FRET efficiencies. Additionally and as already mentioned above, correlation of the basal fluorescence lifetime of mTurquoise2 with the FRET induced changes in fluorescence intensity further helped to discriminate triple-dimeric from trimeric interactions, a strategy that, to our knowledge, has not yet been exploited in microscopic studies.

Due to the fusion protein nature of our FRET constructs, perfect ratios of either 1:1 or 1:1:1 of the respective fluorophores were achieved in the cells. This is a favorable situation for the investigation of FRET which might not be present in protein interaction studies using separate fluorescently labeled proteins. Since FRET is influenced by the donor-acceptor ratio (Chakraborty and Chattopadhyay, 2015; Bajar et al., 2016), we recommend to check for similar expression levels of donor and acceptor fluorophores in the samples and to use reliable controls when applying our method to study protein–protein interactions in living cells.

## Data Availability Statement

The original contributions presented in the study are included in the article/Supplementary Material, further inquiries can be directed to the corresponding author/s.

## Author Contributions

RE carried out experiments and wrote the manuscript. RB devised the project, designed constructs, and wrote the manuscript. Both authors contributed to the article and approved the submitted version.

## Conflict of Interest

The authors declare that the research was conducted in the absence of any commercial or financial relationships that could be construed as a potential conflict of interest.
