# Supplementary material for: A Combined Acceptor Photobleaching and Donor Fluorescence Lifetime Imaging Microscopy Approach to Analyze Multi-Protein Interactions in Living Cells
Source: Front Mol Biosci. 2021 May 14;8:635548. doi: 10.3389/fmolb.2021.635548 (PMC8160235; doi:10.3389/fmolb.2021.635548)
Supplement: Supplementary file 1 [file Data_Sheet_1.docx]

**Supplementary Figure Legends**

**Supplementary Figure 1. Spectral properties of analyzed FRET pairs. A,B,C)** Excitation spectrum (discontinuous line) and emission spectrum (continuous line) of the FRET donor-acceptor pairs mTurquoise2 and YPet (**A**), mTurquoise2 and mCherry (**B**) and YPet and mCherry (**C**). The spectral overlap of the emission spectrum of the donor with the excitation spectrum of the acceptor is marked by the green-striped area.

**Supplementary Figure 2. Influences on donor fluorescence during acceptor photobleaching experiments. A)** Acceptor photobleaching of FRET doublets (TY, TC, YC) and their respective controls (T&Y, T&C, Y&C) (n=5-6). When FRET occurs, the fluorescence intensity of the FRET donor increases after the energy transfer has been abolished by photobleaching of the FRET acceptor. Note the large fluorescence increase of the donor in TY compared to TC and YC. Within the negative controls, T&Y samples showed a slight increase of the donor intensity indicating non-specific FRET, whereas within T&C and Y&C samples the donor intensity remained almost constant. **B)** Calculated absolute FRET efficiencies from acceptor photobleaching experiment (n=5-6). **C)** Investigation of a potential influence of image acquisition during acceptor photobleaching experiments (n=6). Donor fluorescence of TY, TC and YC samples remained constant. **D)** Investigation of a potential photoconversion of YPet or other changes in background fluorescence induced by acceptor photobleaching. After bleaching of YPet (Y) expressed in the absence of mTurquoise2, no changes in blue fluorescence were observed.

**Supplementary Figure 3. Determination of the instrument response function. A)** Average normalized fluorescence decay of fluorescein and fluorescein in presence of a saturated potassium iodide (KI) solution. Iodide ions drastically shorten the lifetime of fluorescein which can be used to determine the instrument response function (IRF) needed for reconvolution fits. In comparison, the calculated IRF suggested by the SymPhoTime software is displayed. **B)** Average fluorescence lifetime of fluorescein (τ = 3.33 ± 0.01 ns) compared to the lifetime of fluorescein in the presence of KI (τ = 0.112 ± 0.0013 ns). Both data sets were fitted with a mono-exponential tail fit (n=5 each).

**Supplementary Figure 4. Evaluation of different fitting models for the determination of the mTurquoise2 lifetime in living HEK293T cells.** **A,B)** FLIM of mTurquoise2 unaffected by FRET (**B**) showing a mono-exponential decay which could be fitted well (Χ²-values close to 1 and only minor random deviation in the residuals) with all selected models. However, slightly improved fitting results were achieved with 2 or 3-exponential decay models. For the mTurquoise2-YPet FRET construct (**A**), the fluorescence decay showed a multi-exponential characteristics consisting of a fast lifetime component directly after the onset of the decay followed by slower lifetime components for the remaining decay process. Rather poor fitting qualities were obtained with 1-exponential decay models (high Χ²-values and a large systematic deviation in the residuals). In contrast, drastically improved fitting results could be achieved by applying either 2- or 3-exponential decay models. **C)** Color-coded fluorescence lifetime images calculated from different fitting models. For the donor mTurquoise2 alone, 3-exponential reconvolution fits failed to process reliable images for the amplitude weighted lifetime. **D)** Average intensity-weighted lifetimes obtained by different fitting models (n=8). For mTurquoise2 unaffected by FRET, lifetimes were determined to be ~4.0ns for every fitting model. For the mTurquoise2-YPet FRET construct, lifetime of mTurquoise2 was reduced to ~2.9ns as assessed by the tail fit and ~2.5ns after applying the reconvolution fits, showing very similar outcomes in both 2- and 3-exponential decay models by using either a measured or a calculated IRF. The inappropriate 1-exponential decay model, however, also gave rise to almost similar lifetimes. **E)** Average amplitude weighted lifetime for multi-exponential fitting models (n=8). For mTurquoise2 unaffected by FRET the lifetimes obtained by 2-exponential models for tail and reconvolution fits were similar at approximately 3.9ns but slightly shorter than the average intensity weighted lifetimes shown in B. In reconvolution fits, 3-exponential models failed to provide reliable amplitude weighted lifetime values for mTurquoise2 alone. In contrast, amplitude lifetime of mTurquoise2-YPet was reduced to ~1.3ns for tail fit, ~1.0ns for reconvolution fits using a calculated IRF and ~ 0.9ns for reconvolution fits using a measured IRF. Although fitting further improved from 2- to 3-exponential models, lifetimes in 3-exponential models were only slightly slower than their counterpart in the respective 2-exponential fitting models.
