# Supplementary figures and images for: A Combined Acceptor Photobleaching and Donor Fluorescence Lifetime Imaging Microscopy Approach to Analyze Multi-Protein Interactions in Living Cells
Source: Front Mol Biosci. 2021 May 14;8:635548. doi: 10.3389/fmolb.2021.635548 (PMC8160235; doi:10.3389/fmolb.2021.635548)

# Comparison of FRET efficiencies obtained by different methods

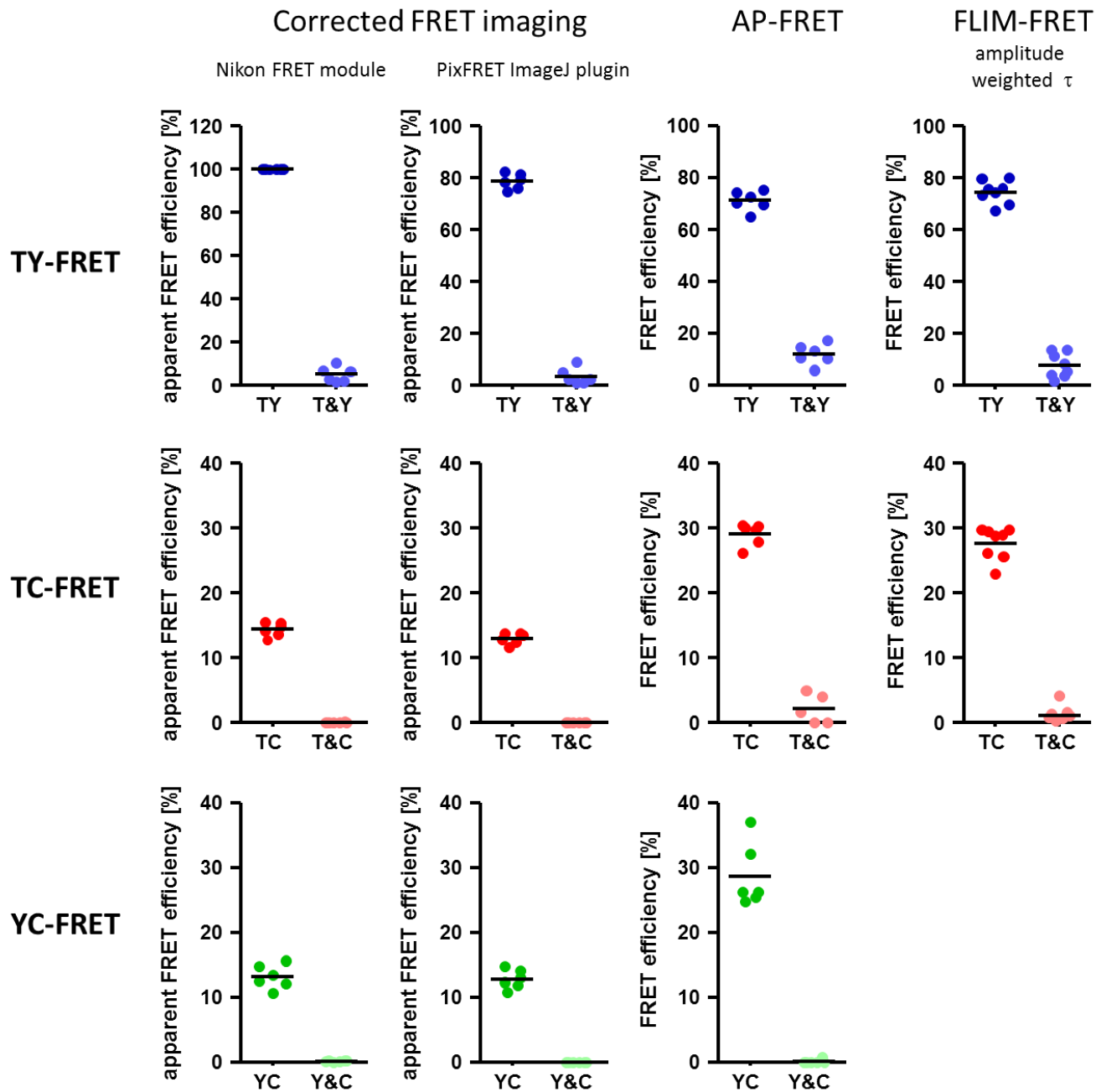

Supplement: Supplementary file 2 [file Data_Sheet_2.pdf]

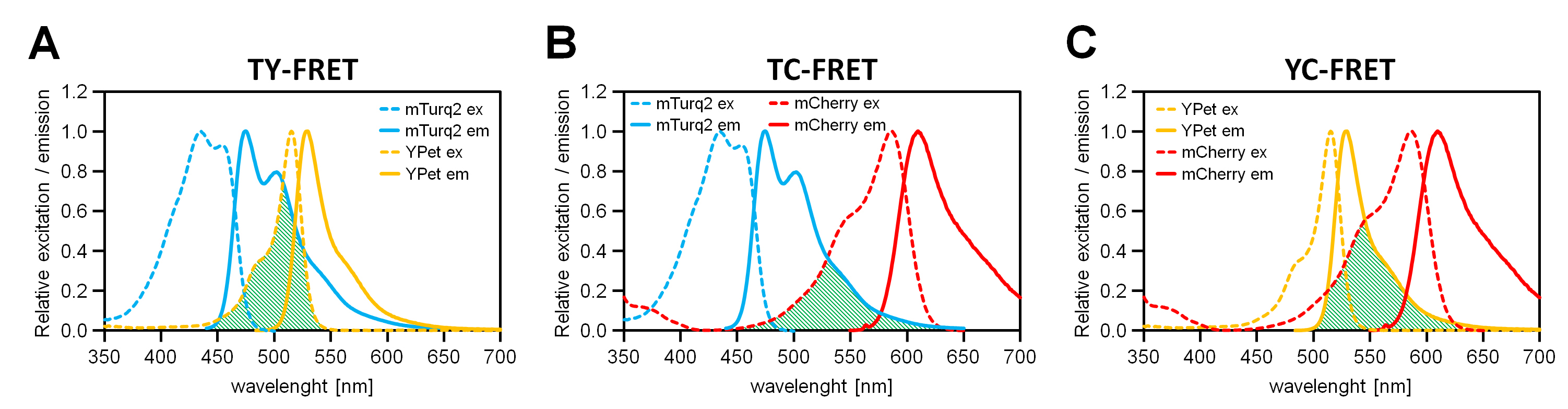

Supplement: Supplementary file 5 [file Image_1.JPEG]

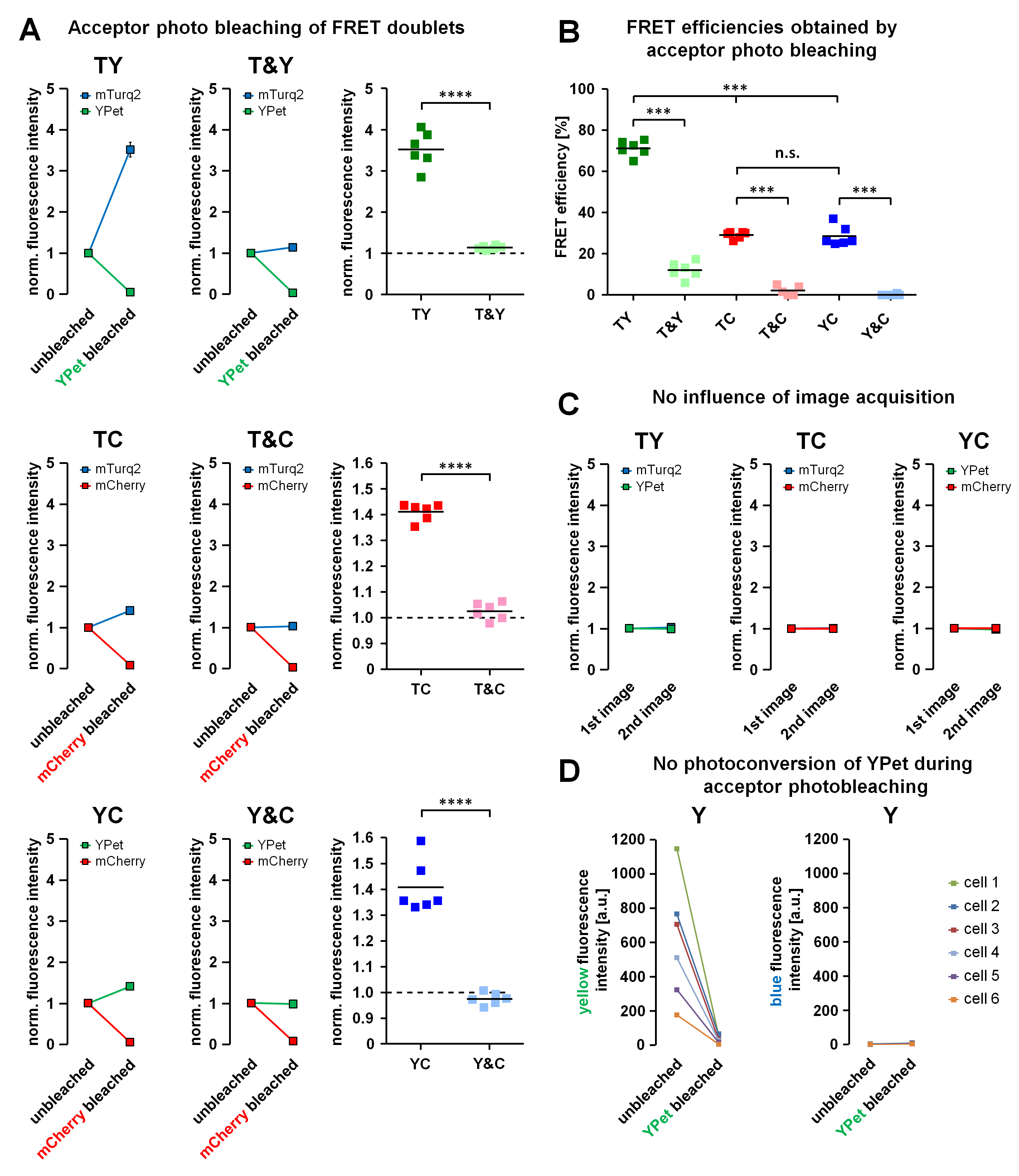

Supplement: Supplementary file 6 [file Image_2.jpg]

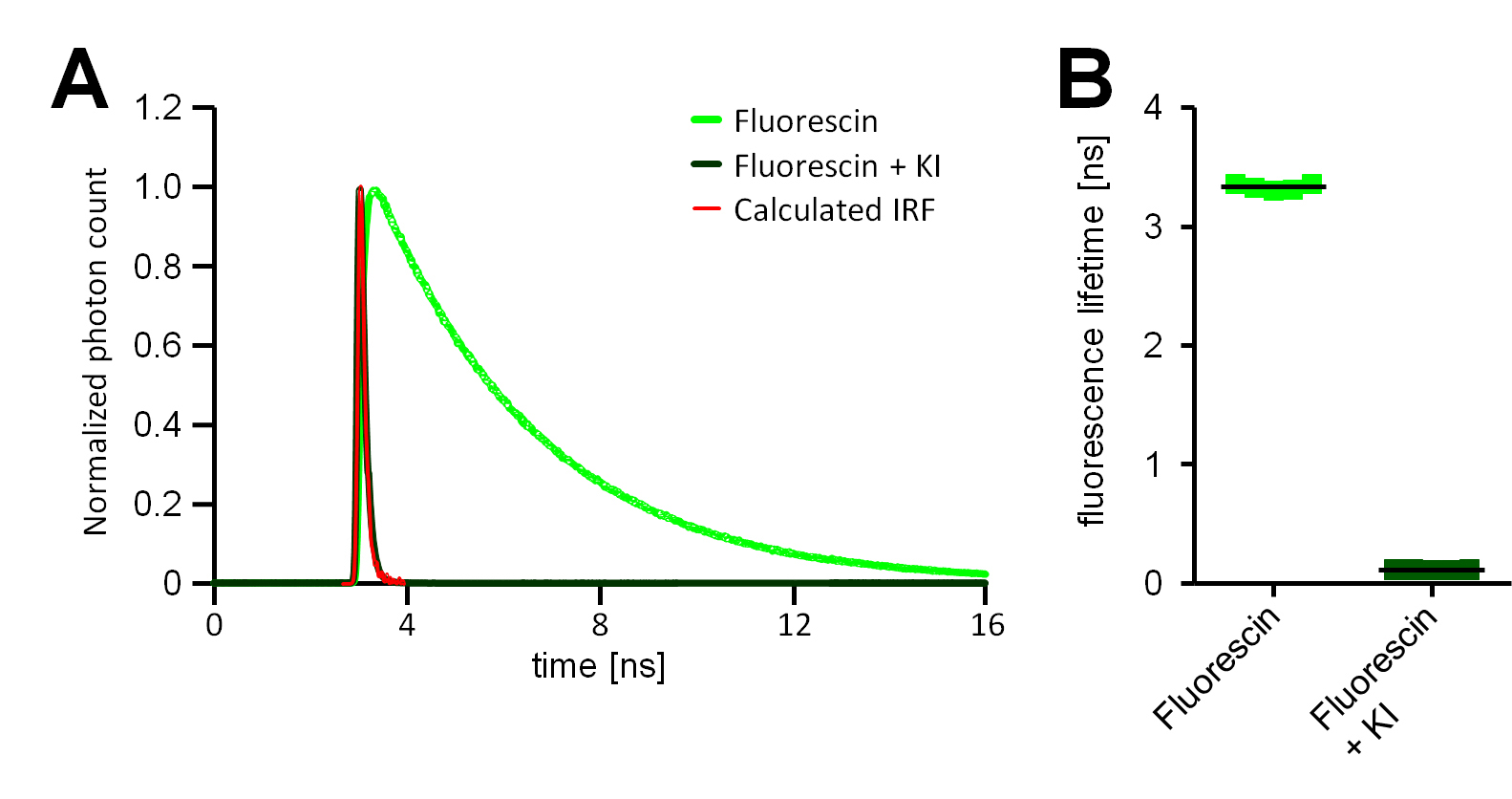

Supplement: Supplementary file 7 [file Image_3.JPEG]

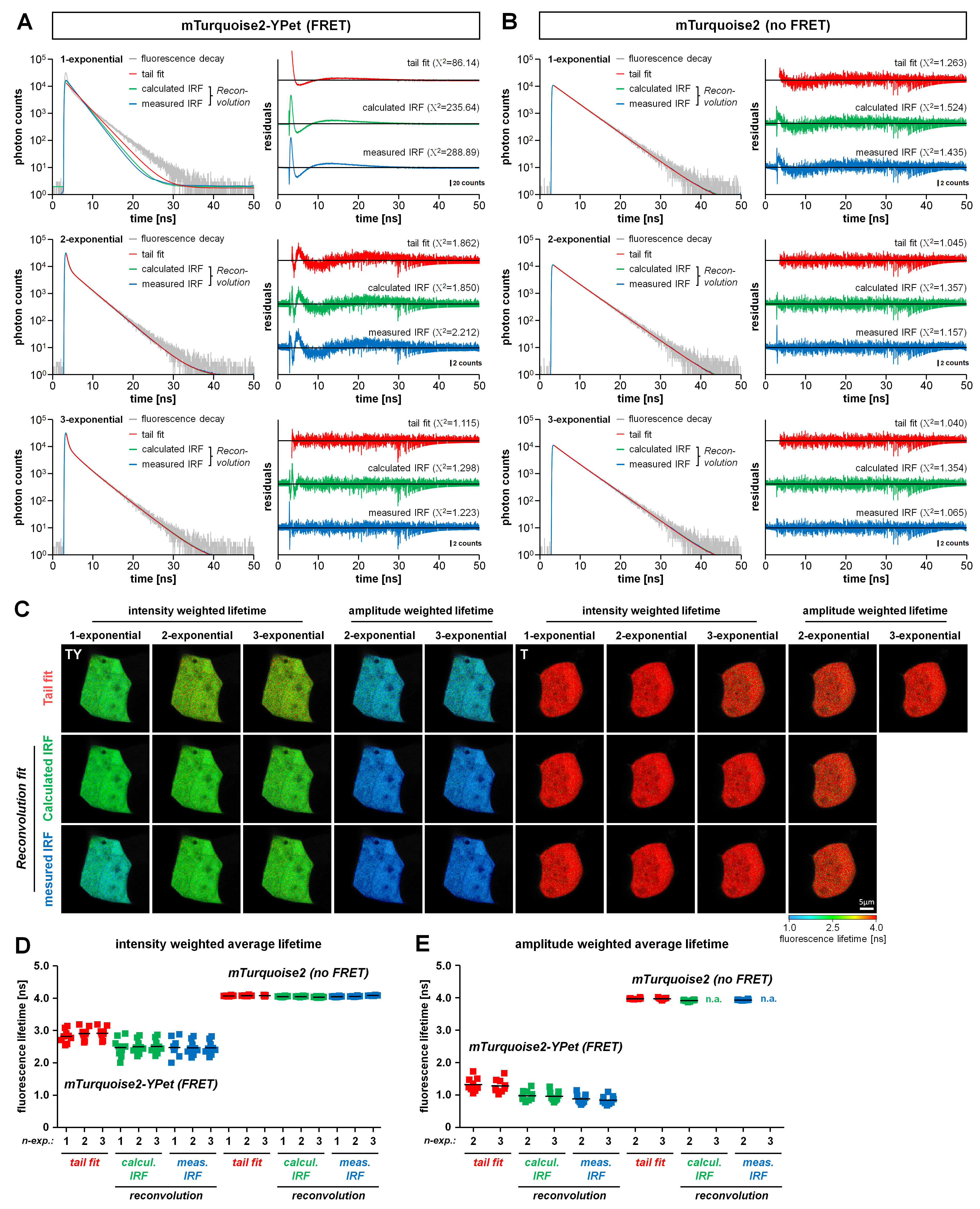

Supplement: Supplementary file 8 [file Image_4.jpg]
