# Supplementary material for: A Combined Acceptor Photobleaching and Donor Fluorescence Lifetime Imaging Microscopy Approach to Analyze Multi-Protein Interactions in Living Cells
Source: Front Mol Biosci. 2021 May 14;8:635548. doi: 10.3389/fmolb.2021.635548 (PMC8160235; doi:10.3389/fmolb.2021.635548)

Non-Specific FRET in the T&Y Combination

Correlation with the donor intensity

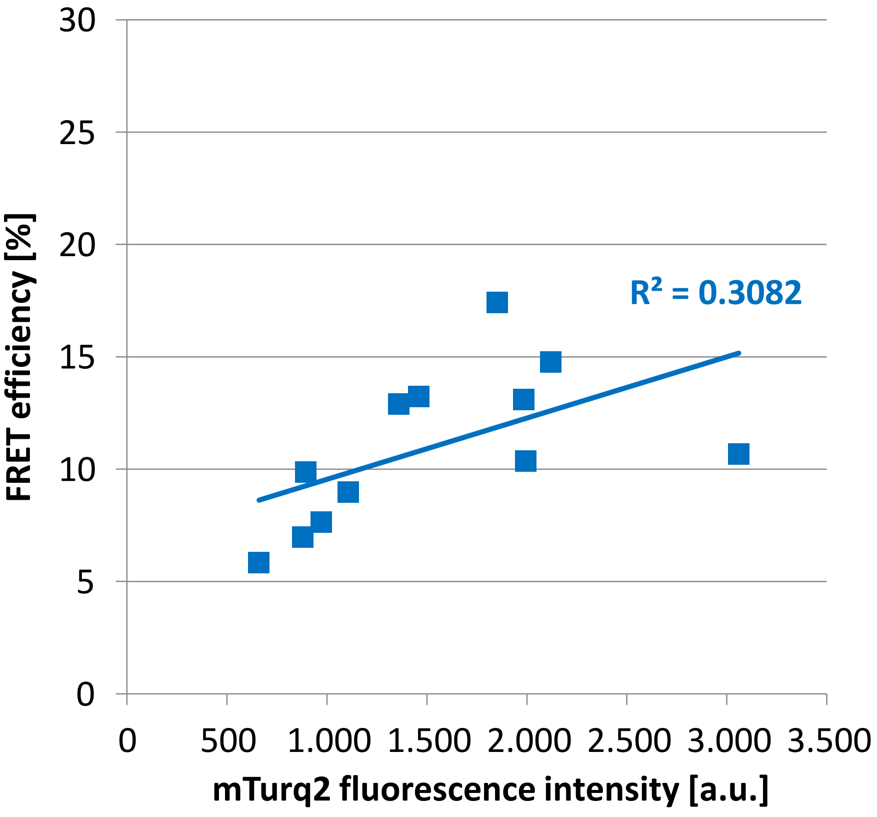

Correlation with the acceptor intensity

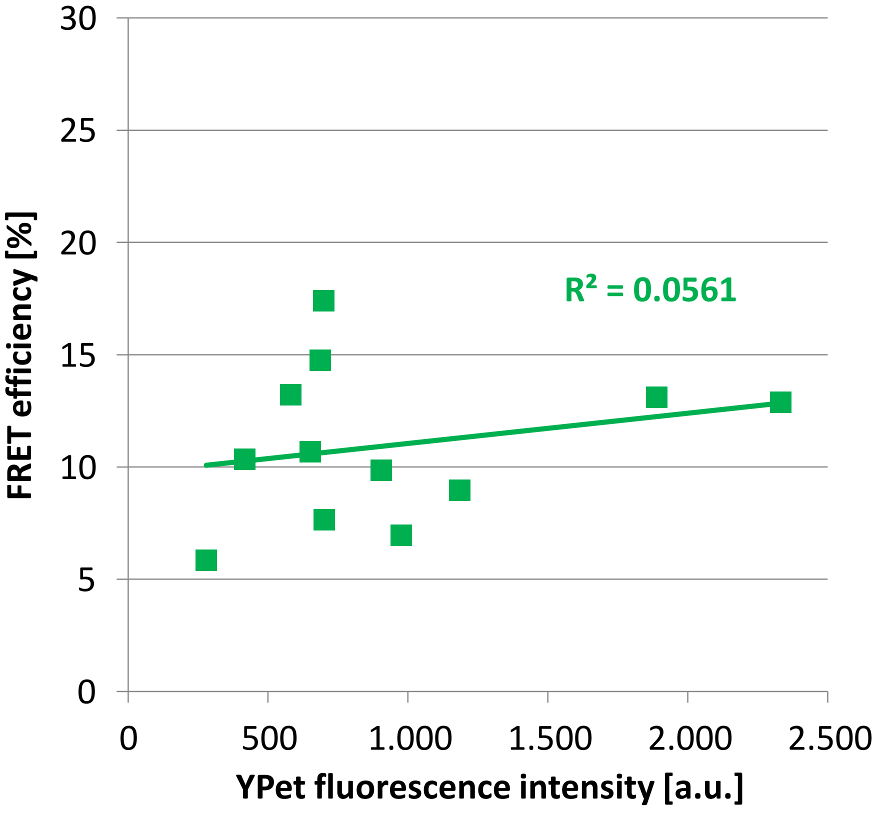

Supplement: Supplementary file 3 [file Data_Sheet_3.pdf]
