# Supplementary material for: A Combined Acceptor Photobleaching and Donor Fluorescence Lifetime Imaging Microscopy Approach to Analyze Multi-Protein Interactions in Living Cells
Source: Front Mol Biosci. 2021 May 14;8:635548. doi: 10.3389/fmolb.2021.635548 (PMC8160235; doi:10.3389/fmolb.2021.635548)
Supplement: Supplementary file 4 [file Data_Sheet_4.pdf]

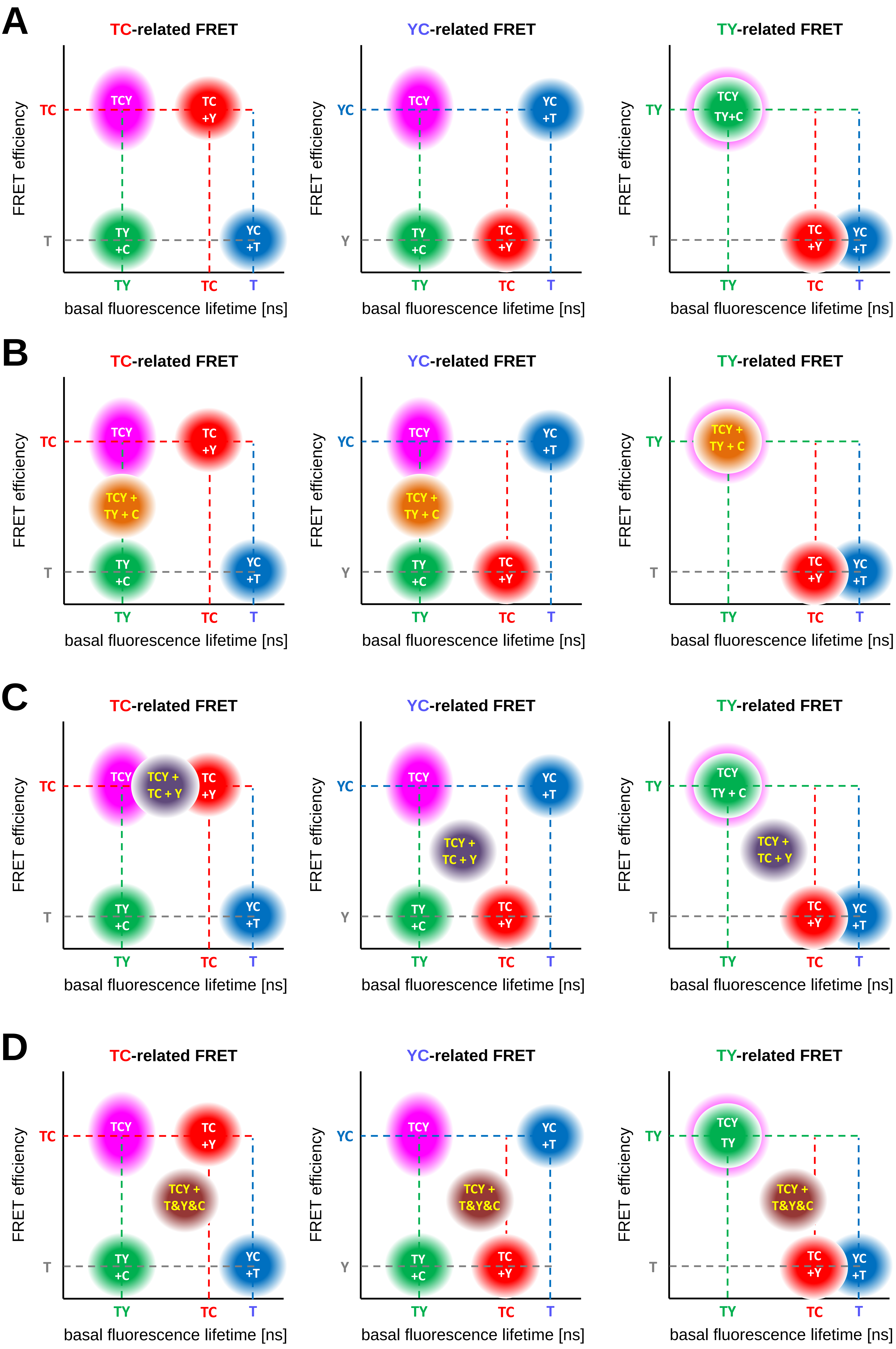
A
TC-related FRET
FRET efficiency
TC
T
TCY
TC
+Y
TY
+C
YC
+T
TY
TC
T
basal fluorescence lifetime [ns]
YC-related FRET
FRET efficiency
YC
Y
TCY
YC
+T
TY
+C
TC
+Y
TY
TC
T
basal fluorescence lifetime [ns]
TY-related FRET
FRET efficiency
TY
T
TCY
TY+C
TC
+Y
YC
+T
TY
TC
T
basal fluorescence lifetime [ns]
B
TC-related FRET
FRET efficiency
TC
T
TCY
TC
+Y
TCY +
TY + C
TY
+C
YC
+T
TY
TC
T
basal fluorescence lifetime [ns]
YC-related FRET
FRET efficiency
YC
Y
TCY
YC
+T
TCY +
TY + C
TY
+C
TC
+Y
TY
TC
T
basal fluorescence lifetime [ns]
TY-related FRET
FRET efficiency
TY
T
TCY +
TY + C
TC
+Y
YC
+T
TY
TC
T
basal fluorescence lifetime [ns]
C
TC-related FRET
FRET efficiency
TC
T
TCY
TCY +
TC + Y
TC
+Y
TY
+C
YC
+T
TY
TC
T
basal fluorescence lifetime [ns]
YC-related FRET
FRET efficiency
YC
Y
TCY
YC
+T
TCY +
TC + Y
TY
+C
TC
+Y
TY
TC
T
basal fluorescence lifetime [ns]
TY-related FRET
FRET efficiency
TY
T
TCY
TY + C
TCY +
TC + Y
TC
+Y
YC
+T
TY
TC
T
basal fluorescence lifetime [ns]
D
TC-related FRET
FRET efficiency
TC
T
TCY
TC
+Y
TCY +
T&Y&C
TY
+C
YC
+T
TY
TC
T
basal fluorescence lifetime [ns]
YC-related FRET
FRET efficiency
YC
Y
TCY
YC
+T
TCY +
T&Y&C
TY
+C
TC
+Y
TY
TC
T
basal fluorescence lifetime [ns]
TY-related FRET
FRET efficiency
TY
T
TCY
TY
TCY +
T&Y&C
TC
+Y
YC
+T
TY
TC
T
basal fluorescence lifetime [ns]

Simulation of correlation plot results assuming uniform ratios for trimeric and dimeric plus monomeric protein states. The characteristic lifetimes (TY, TC, T alone, segmented lines x-axis) and FRET efficiencies (interacting states (TC,YC,TY) and non-interacting states (T,Y), segmented lines y-axis) schematically shown in **A)** define the range of possible positions of the different combinations considered in this analysis. For our simulations, we assumed even ratios (e.g., 1:1:1) for trimeric and dimeric plus monomeric states, which allowed us to predict that FRET efficiencies and lifetimes of fluorescent-protein combinations could be expressed as averages of the respective experimentally determined FRET efficiencies and lifetimes, respectively. **B)** shows the predicted localization of TCY + TY + C in the two-dimensional FRET efficiency versus lifetime plot. Here, donor lifetime and TY-related FRET should be similar to the TY+C values, since TCY and TY+C exhibit similar TY-FRET. However, the TC- and TY-related FRET are assumed to adopt values that are, on average, intermediate between those observed in noninteracting and interacting states, since only the TCY, and not the TY+C combination, allows for TC- and YC-related FRET. **C)** shows the predicted localization of TCY + TC + Y in the two-dimensional FRET efficiency versus lifetime plot. Here, due to the co-occurrence of trimeric and dimeric plus monomeric states, the donor lifetime is assumed to adopt values that are on average between the TY and TC values. The TC-related FRET should be similar to the TC+Y values, since TCY and TC+Y exhibit similar TC-FRET. In contrast, the YC- and TY-related FRET are predicted to adopt values that, on average, lie in between the FRET values of the noninteracting and interacting states, respectively. **D)** shows the localization of TCY + T&Y&C in the two-dimensional FRET efficiency versus lifetime plot. The lifetime is assumed to reach values that lie between the lifetime values of TY and T. In addition, TC-, TY- and YC-related FRET are predicted to adopt values that, on average, are in between the FRET values of the noninteracting and interacting states, respectively.
